# Phytochemical Characterization Using HPLC-DAD, Antiglycation Effect at Multiple Stages, Anti-Inflammatory and Analgesic Activities of *Solanum elaeagnifolium* Cav: Experimental and Computational Studies

**DOI:** 10.3390/cimb48080810

**Published:** 2026-08-11

**Authors:** Mohammed Bouslamti, Rhizlan Abdnim, Rafik El-Mernissi, Otmane Zouirech, Salah-eddine Chebaibi, Moneerah J. Alqahtani, Jawaher H. Alqahtani, Joe Miantezila Basilua, Lhoussain Hajji, Naoufal El Hachlafi, Ahmed Samir Benjelloun

**Affiliations:** 1 Laboratory of Biotechnology, Conservation and Valorisation of Bioresources (BCVB), Faculty of Sciences Dhar El Mahraz, University Sidi Mohamed Ben Abdellah, B.P. 1796, Fez 30050, Morocco; 2 Laboratory of Bioresources, Biotechnology, Ethnopharmacology, and Health, Faculty of Sciences, Mohammed First University, B.P. 717, Oujda 60000, Morocco; 3 Bioactive and Environmental Health Laboratory, Faculty of Sciences, Moulay Ismail University, B.P. 11201, Meknes 50070, Morocco; 4 Physiology and Physiopathology Team, Faculty of Sciences, Genomic of Human Pathologies Research Centre, Mohammed V University, B.P. 1000, Rabat 10106, Morocco; 5 Plant Biotechnology Team, Faculty of Sciences, Abdelmalek Essaadi University, Tetouan 93002, Morocco; 6 Department of Pharmacognosy, College of Pharmacy, King Saud University, P.O. Box 2457, Riyadh 11451, Saudi Arabia; 7 Department of Biostatistics and Mathematics, Faculty of Pharmacy, Paris Cité University, 4, avenue de l’Observatoire, 75006 Paris, France; 8 Faculty of Medicine and Pharmacy, Ibn Zohr University, Guelmim 80000, Morocco

**Keywords:** *Solanum elaeagnifolium*, glycation products, HPLC-DAD, anti-lipoxygenase, docking study, SDG3, BSA-Denaturation, analgesic

## Abstract

*Solanum elaeagnifolium* has been utilized for its analgesic, anti-inflammatory, and antioxidant properties to treat a range of conditions, including pain and inflammation. It possesses antibacterial, insecticidal, and molluscicidal properties as well. To investigate the pharmacological potential of the *S. elaeagnifolium* extract, in vitro, in vivo, and in silico studies were used. Albumin denaturation, heat-induced anti-haemolytic action, and lipooxygenase inhibition were the three techniques used to test anti-inflammatory effectiveness. The phenolic chemicals utilized were identified through the use of high-performance liquid chromatography (HPLC). The effectiveness of the extract in lowering problems connected to diabetes was further evaluated by evaluating fructosamines, carbonyl groups, and β-amyloid formations in albumin glycation. Regarding in vivo studies, the Writhing test and the tail flick test were the two techniques used to evaluate analgesic activity. For docking analysis, we used the following identifiers: cyclooxygenase (PDB ID: 6COX), lipooxygenase (PDB ID: 3V99) and VC1 domain of the receptor for advanced glycation products (PDB ID: 7LMW). The most prevalent phenolic chemicals, according to HPLC analysis, were 3,4-dihydroxybenzoic acid (4.78%), caffeic acid (1.28%), gallic acid (8.18%), ursolic acid (2.6%), syringic acid (1.78%), quercetin (24.09%), catechin (6.67%), and p-coumaric acid (20.49%). Significant suppression of albumin glycation and fructosamine production was demonstrated by the extract, indicating that it may help lessen difficulties associated with diabetes. Furthermore, the extract demonstrated a strong anti-inflammatory impact, with an IC_50_ of 146 ± 1.17 μg/mL for lipooxygenase inhibition, 87.64 ± 5.35 μg/mL for anti-haemolytic action, and 86.94 ± 1.63 μg/mL for albumin denaturation inhibition. A similar analgesic effect to those of analgesic medications was also demonstrated by SEFE extract. Reinforcing the relevance of the observed effects, the in silico study of the tested activities validated these findings. These findings indicate significant pharmacological promise in the management of diabetes consequences, including inflammation and protein glycation, as well as an analgesic.

## 1. Introduction

Historically, *Solanum elaeagnifolium* was used as an antiseptic for toothaches, gastrointestinal problems, and sore throats; it was also used to treat snakebites, ear inflammations, stomach pains, and angina. Because of its many biological properties, *S. elaeagnifolium* can be investigated in a variety of medical, agricultural, and environmental contexts [1]. First, it has analgesic qualities that can alleviate pain associated with a number of conditions, and its anti-inflammatory qualities help reduce inflammation in the body, relieving the symptoms of inflammatory diseases; and, thanks to its potent antioxidant properties, it can help shield cells from oxidative damage, thereby preventing a number of diseases [2,3,4]. Additionally, *S. elaeagnifolium* has molluscicidal and insecticidal chemicals, which makes it a useful ally in the battle against agricultural pests including dangerous insects and mollusks. Insect-borne disease vectors can be controlled by using it as a larvicide [5,6]. This plant has antimicrobial qualities that can be used medicinally to cure illnesses by preventing the growth of harmful microbes like viruses, fungi, and bacteria [2,4,7]. Therapeutically, this herb contains antidiabetic and anti-hyperlipidemic effects. *S. elaeagnifolium*, finally yet importantly, has anticancer and chemopreventive qualities that may help treat existing tumors and prevent some forms of cancer. The phytochemical composition of *Solanum elaeagnifolium* is rich and diverse, dominated by two major families of secondary metabolites, namely phenolic compounds and steroidal alkaloids, with notable variations depending on the plant organs. The leaves are particularly concentrated in polyphenols, among which flavonoids occupy a preponderant place, with quercetin, kaempferol and their glycosylated derivatives such as rutin and quercetin-3-O-β-D-glucoside being the major representatives, alongside phenolic acids such as 4-hydroxybenzoic acid and trans-3-hydroxycinnamic acid. In parallel, this solanaceous plant synthesizes steroidal alkaloids typical of its family, essentially solasonine, solanidine, solasodine and β-solamargine, the latter being able to represent up to nearly 80% of the total alkaloid fraction in certain extracts, while diosgenin, a steroidal sapogenin, is also detected [8,9,10].

Today, a wide range of ailments, including cancer, immunological problems, and infections, are treated with numerous herbs that were once employed in traditional medicine. Numerous plants used in traditional medicine have been shown to have anti-inflammatory and antioxidant properties in vitro and in vivo, and studies have also shown how various natural compounds isolated from these plants work. By preventing pro-inflammatory cytokine production or inflammatory cell activation, for instance, these active substances can affect the inflammatory response at various stages [11,12,13,14,15,16,17].

Reactive sugars, including glucose, attach to proteins non-enzymatically in a process known as protein glycation, producing advanced glycation products (AGEs). The main cause of this syndrome is elevated blood glucose, which is typical among diabetics. Proteins can have their structure and function changed by these AGEs, which reduces their effectiveness and causes cellular malfunction. Furthermore, AGEs can build up in tissues, impacting organs including the kidneys, heart, and eyes. They are also linked to a variety of diseases, including diabetes and cardiovascular disease [18,19,20].

The production of AGEs is frequently associated with inflammation because these products have the ability to activate particular cell receptors called RAGE receptors (receptors for advanced glycation products) [21]. A chronic inflammatory response is triggered by the production of inflammatory cytokines when AGEs attach to these receptors. This activation can exacerbate the body’s inflammatory processes, which can lead to the emergence of a number of inflammatory disorders. Therefore, the development and maintenance of inflammation are significantly influenced by protein glycation and AGE formation, which highlights the health hazards, especially when it comes to metabolic illnesses [22,23].

This study sought to determine whether the hydroethanol extract of *S. elaeagnifolium* (SEFE) leaves could help avoid problems from diabetes, specifically by looking at its anti-glycation qualities. At each stage, the study assessed several glycation markers: fructosamines for the first stage, protein carbonyls for the middle stage, and AGEs for the last stage. Using amyloid-specific dyes like Congo red, the study also looked at albumin aggregation. Identifying the chemical profile and anti-inflammatory actions in vitro was another goal.

## 2. Material and Methods

### 2.1. Plant Material

*S. elaeagnifolium* is a member of the Solanum genus and the Solanaceae family. In November 2023, leaf harvesting occurred in the Moroccan city of Fez (34°04′04.2 N, 5°01′26.4 W), where plant specimens were gathered. Professor Bari Amina of Sidi Mohamed Ben Abdellah University’s Department of Life Sciences identified the species (Voucher number: E17/14054).

### 2.2. Hydroethanolic Extract

The leaves of *S. elaeagnifolium* were first dried in the dark for seven days at a constant temperature of 25 °C, then ground into a fine powder and stored in airtight plastic containers. The hydroethanolic extract (SEFE) was prepared by macerating 50 g of this powder in 500 mL of an ethanol/water mixture (70/30, *v*/*v*) under magnetic stirring at 150 rpm, at room temperature (25 ± 2 °C), for exactly 72 h. The macerate was then filtered through Whatman No. 1 filter paper, and the filtrate was concentrated under vacuum using a rotary evaporator at 40 °C. The yield of the dried extract was 14.8% (*w*/*w*) based on the initial dry plant material, and the final extract was stored at –20 °C until further use.

### 2.3. HPLC-DAD

The sample and standard solutions were prepared at a concentration of 30 mg/mL and then passed through a 0.4 µm membrane filter to remove particles. Chromatographic analysis was performed using HPLC-DAD. The analysis was performed on a Kinetex C18 column in reversed-phase mode (250 × 4.6 mm, with particle size 2.6 µm). A binary gradient separation was employed, using a mobile phase of 0.1% acetic acid in water (solvent A) and methanol (solvent B). The gradient profile was as follows: from 0 to 3 min, 5–25% B; from 3 to 6 min, isocratic at 25% B; from 6 to 9 min, 25–37% B; from 9 to 13 min, isocratic at 37% B; from 13 to 18 min, 37–54% B; from 18 to 22 min, isocratic at 54% B; from 22 to 26 min, 54–95% B; from minute twenty-six to minute twenty-nine, isocratic at ninety-five per cent B. The chromatographic system was operated at a flow rate of 1 mL/min, maintaining the column at a temperature of 30 °C. UV-Vis identification was carried out in a spectral range of 200 to 400 nm, with the chromatographic profiles observed at 280 nm. Compounds were identified by comparing their retention times with those of authentic standards [24].

### 2.4. Antiglycation Effect of Albumin at Different Stages

The glycation of albumin was performed using a slightly modified version of the procedure outlined by Bouchmaa et al. (2022) [25]. In short, a 100 mM potassium phosphate-buffered solution (pH 7.4, containing 0.2% sodium azide) was used to make a 10 mg/mL BSA solution. Next, 1 mL of this BSA solution was combined with a 500 mM glucose solution. SEFE and aminoguanidine (AG) were added to the mixture, which was then incubated at 37 °C in the dark for a week. The fluorescence of advanced glycation end products (AGEs) was measured using a spectrofluorometer with excitation and emission wavelengths set at 355 nm and 460 nm, respectively. The results were calculated using the formula provided below and expressed as a percentage of inhibition.

#### 2.4.1. Estimation of Fructosamine

Fructosamine production was evaluated using the thiobarbituric acid (TBA) assay [26]. TBA (0.05 M) was added to glycated BSA, and the mixture was heated in a water bath for 20 min before being allowed to cool to room temperature. At 443 nm, absorbance was measured, and the following formula was used to get the fructosamine % inhibition:

$$Inhibition\;\left(\%\right)=\frac{\left(Ac-As\right)}{Ac}\times 100$$

where Ac represents the absorbance of the positive control group, and As represents the absorbance after adding the extract sample.

#### 2.4.2. Estimation of Carbonyl Groups

To measure the carbonyl groups in albumin, we employed the technique outlined by Safari et al. (2018) [27]. In short, 0.5 mL of glycated samples were combined with 10 mM DNPH in 2.5 M HCl in glass tubes, and the mixture was then allowed to sit at room temperature for an hour. The carbonyl concentration was computed using the molar absorptivity of DNPH (365 nm = 21 mM-cm) and absorbance was measured at 365 nm. The percentage of inhibition was calculated as follows:

$$Inhibition\;\left(\%\right)=\frac{\left(Ac-As\right)}{Ac}\times 100$$

where Ac represents the absorbance of the positive control group, and As represents the absorbance after adding the extract sample.

#### 2.4.3. Estimation of Amyloid β-Structures

The binding between Congo red and β-amyloid aggregation in the samples was determined by being able to quantify its absorbance at 530 nm, as detailed in research conducted by Rashmi S. Tupe et al. in 2015 [28]. Therefore, 100 μL of Congo red and 0.5 mL of glycated albumin were mixed and incubated for 20 min at normal temperature. A wavelength of 530 nm was used to quantify absorbance. The equation below was used to calculate the percentage inhibition:

$$Inhibition\;\left(\%\right)=\frac{\left(Ac-As\right)}{Ac}\times 100$$

where Ac represents the absorbance of the positive control group, and As represents the absorbance after adding the extract sample.

### 2.5. Anti-Inflammatory In Vitro

#### 2.5.1. Inhibition of Albumin Denaturation

The in vitro anti-inflammatory activity of the extracts was measured using an albumin denaturation assay, based on literature references. Briefly, a 15 min incubation at 37 °C was performed with 0.5 mL of a 0.2% (*w*/*v*) bovine serum albumin (BSA) solution in Tris buffer, pH 6.8, combined with various concentrations of plant extract or diclofenac sodium (positive control). The mixtures were then heated to 72 °C for 5 min to denature the proteins [29]. The absorbance of the solutions produced was assessed at 660 nm to determine the amount of protein aggregation. All experiments were carried out in triplicate. Using the following formula, the proportion of protein denaturation was determined:

$$Inhibition\;\left(\%\right)=\frac{\left(Ac-As\right)}{Ac}\times 100$$

where Ac represents the absorbance of the positive control group, and As represents the absorbance after adding the extract sample.

#### 2.5.2. Preparation of a Suspension of Erythrocytes

Following the collection of blood from a healthy person, the blood is drawn into heparin tubes and centrifuged for ten minutes at 3000 rpm. Physiological water is used to wash the pellet three times in succession, while the plasma is disposed of. The suspension of erythrocytes is diluted to 10% [30].

#### 2.5.3. Heat-Induced Hemolysis

Heat-induced hemolysis was evaluated using the Gunathilake et al. technique [31]. Using this technique, 2.95 mL of phosphate buffer (pH 7.4) and 0.05 mL of a blood cell solution were mixed in triplicate with different extracts. In a shaking water bath, the produced mixture was incubated for 20 min at 54 °C. The mixture was centrifuged for three min at 2500 rpm after incubation. A UV/VIS spectrometer was then used to detect the supernatant’s absorbance at 540 nm. A buffer solution containing phosphate served as the experiment’s control. The reference standard was aspirin.

#### 2.5.4. Lipoxygenase Inhibition

The in vitro anti-inflammatory activity of the leaf extract was determined by the Lipoxygenase Inhibition (5-LOX) technique, following the oxidation of linoleic acid at 234 nm as described elsewhere. Briefly, 20 µL of the sample (dissolved in ethanol) and 20 µL of glycine max 5-LOX (100 U/mL) were first mixed with 0.2 mL of phosphate buffer (0.1 M, pH 9) and the solution incubated at 25 °C for 6 min. Next, 20 µL linoleic acid (4.18 mM in ethanol) was added to the mixture and monitored for 3 min at 234 nm [14,32].

### 2.6. Analgesic Activity

#### 2.6.1. Animals

Male rats weighing about 250 g served as the animals. Sidi Mohammed ben Abdallah University’s Faculty of Sciences in Fes, Morocco, has an animal facility where these animals were acquired. Every animal was kept in a cage at room temperature (25 °C) with constant access to food and water, and they were all on a 12 h light/12 h dark cycle. For the care and use of animals, all relevant institutional, national, and/or international guidelines were adhered to. The reference number L.20. USMBA-SNAMOPEQ 2023-03 is used to document this approval.

#### 2.6.2. Writhing Test

Rats weighing 180–200 g were independently assigned to four groups of six rats each: Group 1 (control) received 0.9% saline treatment, Group 2 received aspirin pretreatment (100 mg/kg body weight), Group 3 received SEFE (100 mg/kg body weight), and Group 4 received SEFE (150 mg/kg body weight). Thirty min after the treatments, 3.75 mL/kg of 3% acetic acid solution was injected intraperitoneally to cause contortions. The number of contortions was counted during a 10 min observation period following the acetic acid injection [33]. The inhibition (%) of abdominal contractions was assessed as follows:

$$Inhibition\left(\%\right)=(1-\left(\;\frac{Nt}{Nc}\right))\times 100$$

where Nt and Nc represent the number of contortions observed in the treatment and control groups respectively.

#### 2.6.3. Tail Flick Test

The test was conducted on four groups of Wistar albino rats weighing between 180 and 200 g. Six rats were included in each group. SEFE was administered orally to the third group at a dose of 150 mg/kg. The first group was seen as the control, while the second group served as a reference (100 mg/kg of aspirin). A cut-off time of 20 s was maintained while recording the tail reaction (ANALGESY, METER LE 7106 (Panlab, a brand of Harvard Apparatus, Barcelona, Spain)), which is the radiant heat sensation from the spine. Each batch underwent the tail test prior to treatment as well as 30, 60, 90, and 120 min following drug delivery [34].

### 2.7. Molecular Docking

#### 2.7.1. Ligand Preparation

From the PubChem database, all phytocompounds identified by HPLC in the plant were obtained in SDF format. The LigPrep module of Maestro V. 11.5 from the Schrödinger suite was used to prepare ligands with optimization with the OPLS3 force field. For each compound, the ionization state was adjusted to a pH of 7.0 ± 2.0, allowing for the generation of up to 32 potential stereoisomers per molecules [35,36].

#### 2.7.2. Protein Preparation

Proteins selected for docking analysis were obtained from the Protein Data Bank. The Protein Data Bank (www.rcsb.org) was used to obtain proteins selected for docking analysis with the following identifiers: cyclooxygenase (PDB ID: 6COX), lipoxygenase (PDB ID: 3V99), and VC1 domain of the receptor for advanced glycation end products (PDB ID: 7LMW). The preparation of these protein structures required multiple improvements: addition of hydrogen atoms, correction of bond orders, removal of water molecules, assignment of hydrogen bonds, optimization of receptor atom charges, and energy minimization using the OPLS3 force field [4,37].

#### 2.7.3. Glide Standard Precision (SP) Ligand Docking

Ligand docking was conducted using the Standard Precision (SP) mode of Glide, integrated within Schrödinger-Maestro version 11.5. During the process, penalties were applied to amide bonds that deviated from cis/trans conformations. Van der Waals interactions involving ligand atoms were scaled down to 0.80, with a partial charge cutoff of 0.15. The docking outcomes were evaluated based on the glide score, derived from the energy-minimized ligand poses. The pose with the lowest glide score was selected as the optimal docking result for each ligand [36].

## 3. Results and Discussion

### 3.1. HPLC Analysis

**Figure 1:**
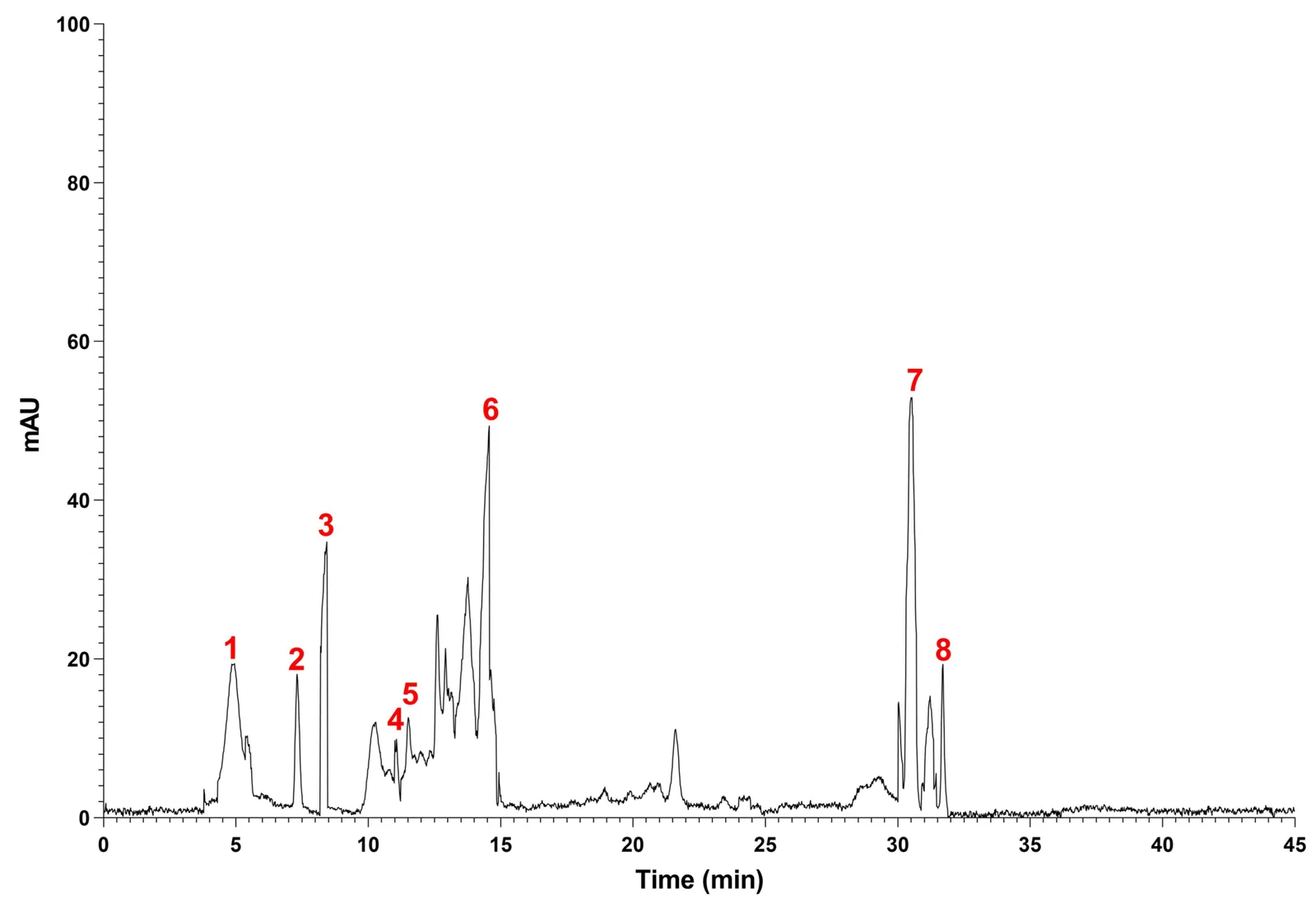
HPLC-DAD chromatogram of *S. elaeagnifolium* leaf extract (SEFE), Gallic acid (1), 3-4-dihydroxybenzoic acid (2), catechin (3), Caffeic acid (4), Syringic acid (5), P-coumaric acid (6), quercetin (7), Ursolic acid (8).

There are eight polyphenolic components in the hydroethanol extract of *S. elaeagnifolium* (Table 1) (Figure 1). Among the chemical compounds found were flavonoids and polyphenols. The primary phenolic chemicals found were quercetin (24.09%), ursolic acid (2.6%), syringic acid (1.78%), catechin (6.67%), caffeic acid (1.28%), gallic acid (8.18%), P-coumaric acid (20.49%), and 3-dihydroxybenzoic acid (4.78%). Numerous advantageous biological actions are displayed by the polyphenolic chemicals found in the hydroethanol extract of *S. elaeagnifolium*. Of them, quercetin, a significant flavonoid, is known for its anti-inflammatory, anti-cancer, and antioxidant qualities, which help to lower inflammation and protect the heart [38,39,40]. Ursolic acid is a triterpenoid that has anti-inflammatory and anti-cancer characteristics, as well as the ability to protect the liver and regulate lipid metabolism [41,42]. Both caffeic acid and syringic acid have anti-inflammatory and antioxidant qualities [43,44,45]. They also help to regulate the immune system and guard against neurodegenerative illnesses. The flavonoid catechin, which has strong antioxidant qualities, enhances blood flow and has anti-cancer benefits [46,47,48,49].

### 3.2. Antiglycation Effect of Albumin at Different Stages

#### 3.2.1. Effect of SEFE on Multiples Stages of Albumin Glycation

The data showcased in Table 2 demonstrates that SEFE significantly inhibits the albumin glycation in all three stages: The early glycation product fructosamine that was used as the representative glycation compounds (TBA) were found to be inhibited by SEFE with IC_50_ values 0.51 ± 0.012 and 0.45 ± 0.006 mg/mL of SEFE and AG (standards control), respectively. While in the case of protein carbonyl compounds, which serve as intermediate stage markers (DNPH), inhibition was observed with IC_50_ value of 0.52 ± 0.005 and 0.46 ± 0.003 mg/mL of SEFE and AG respectively. Fluorescence analysis of advanced glycation end-products (AGEs) in the later stages, suggested that SEFE possesses inhibitory properties against them with IC_50_ value of 0.49 ± 0.007 and 0.45 ± 0.005 mg/mL of SEFE and AG respectively. The inhibition mode exhibits a dependence on the dosage, with the most effective dose being 1 mg/mL for each extract.

#### 3.2.2. Effect of SEFE on β-Aggregation During Glycation

**Figure 2:**
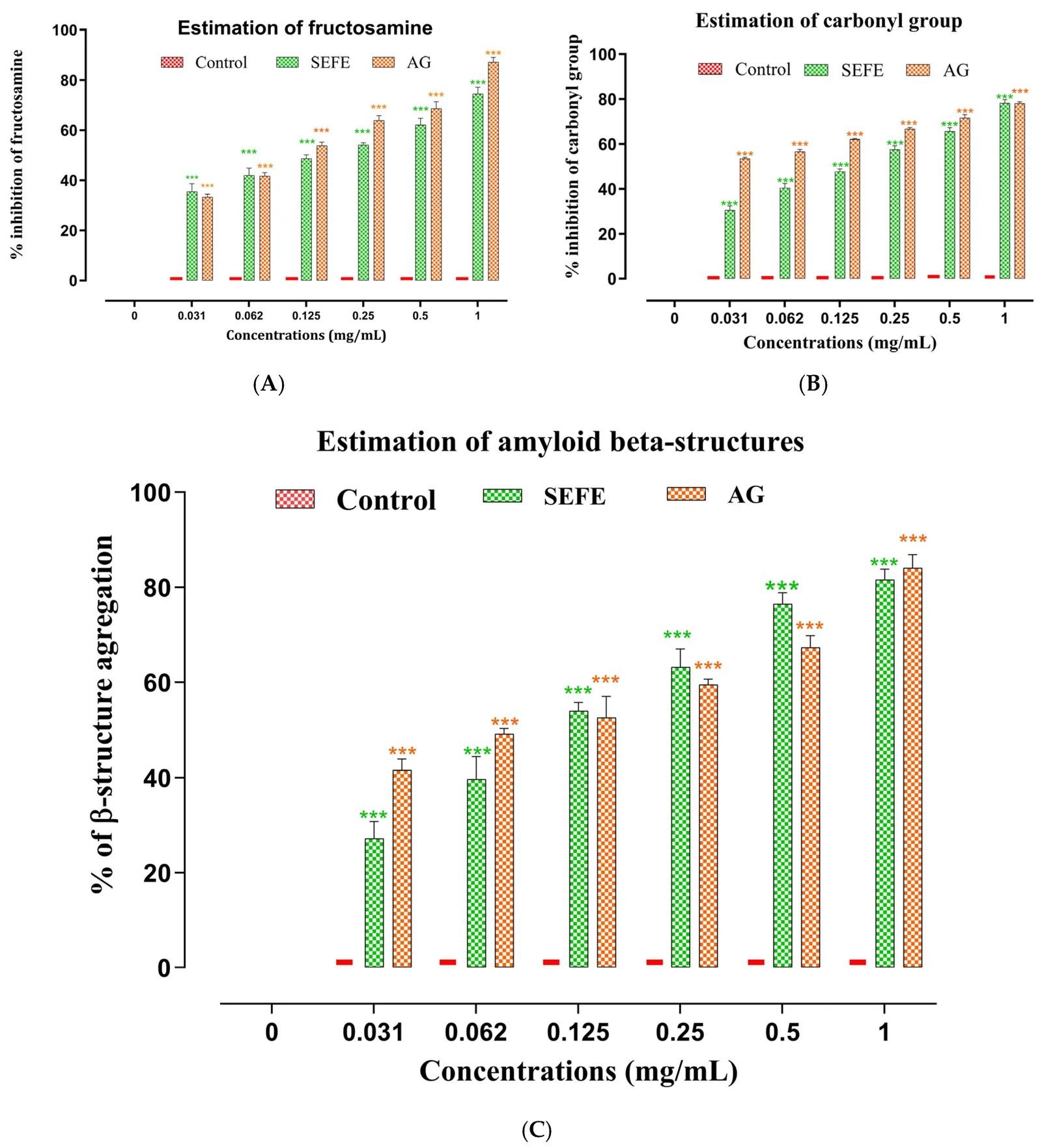
Impact of SEFE extract and AG (1 mg/mL) on the alteration of albumin glycation, concerning (**A**) inhibition of fructosamine, (**B**) inhibition of protein carbonyls, (**C**) β aggregation inhibition. Values are expressed as mean ± standard deviation (SD). *** *p* < 0.0001 compared to the control group.

Glycation represents a pivotal mechanism that induces alterations in protein conformation by elevating the levels of amyloid cross β-structures. These structures play a fundamental role in protein aggregation. We conducted an investigation to assess how SEFE can hinder the aggregation of glycated albumin, a process linked to amyloidosis, employing Congo red amyloid markers. Figure 2C illustrated that the inclusion of SEFE exhibited strong inhibition alongside the amyloid marker. The IC_50_ value demonstrated in Table 3 shown a strong inhibition activity of amyloid cross β-structure by SEFE and AG with 0.45 ± 0.006 and 0.46 ± 0.006 mg/mL. One important element in the development of advanced glycation products (AGEs), oxidative stress, may be lessened by 3-dihydroxybenzoic acid. Therefore, it may help avoid problems related to protein glycation, especially those related to diabetes, by reducing its production [18,26].

Similarly, gallic acid, a hydrolyzable tannin, inhibits protein glycation and possesses strong antioxidant qualities. By restricting the oxidation of reducing sugars, it stops advanced glycation products from forming. Especially in situations when oxidative stress is elevated, like diabetes, its function in lowering blood sugar levels may have significant consequences for preventing age-related and metabolic problems [50,51]. By decreasing the oxidation of sugars and preventing the initial phases of the Maillard reaction, quercetin has been demonstrated to prevent protein glycation. Quercetin helps lower advanced glycation products by altering the cellular response to oxidative stress, which may be very helpful in the treatment of challenges associated with diabetes and aging [39,51].

### 3.3. Anti-Inflammatory In Vitro

#### 3.3.1. Inhibition of Albumin Denaturation

**Figure 3:**
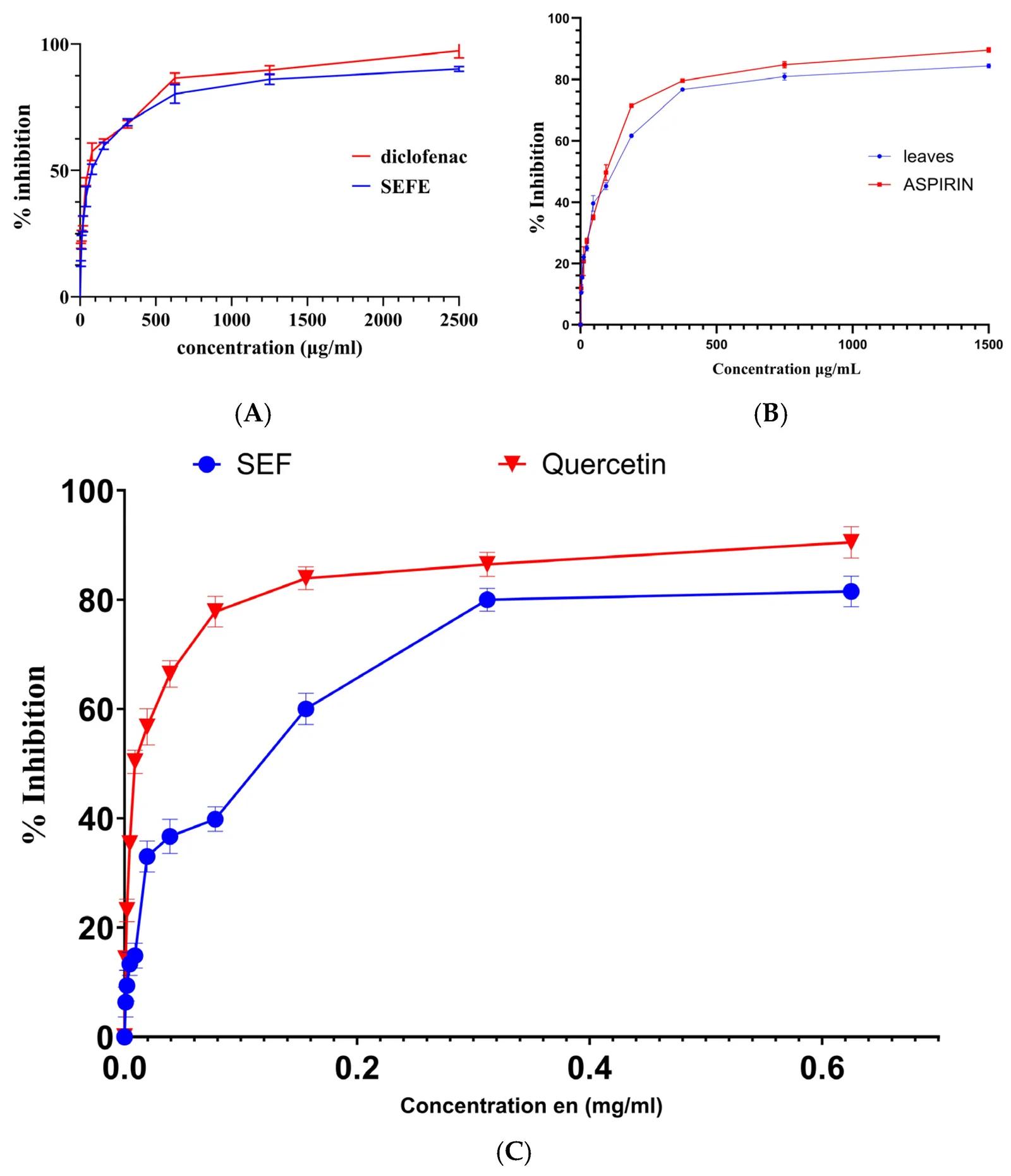
Anti-inflammatory effect in vitro, (**A**) inhibition of SEFE and diclofenac on BSA denaturation, (**B**) impact of aspirin and SEFE on the prevention of haemolysis in heat-induced tests, (**C**) the proportion of LOX inhibition for SEFE and the positive control quercetin.

Figure 3A shows the percentage inhibition of BSA denaturation that was achieved after the anti-inflammatory effects of SEFE and diclofenac on BSA denaturation were assessed. The results show that the SEFE extract has an inhibition lower than that of the positive control, diclofenac (*p* < 0.01), with IC50 values of 86.94 ± 1.63 and 76.46 ± 1.94 μg/mL, respectively. When proteins are subjected to external stressors or substances like strong acids or bases, concentrated inorganic salts, organic solvents, or heat, they lose their tertiary and secondary structures, a process known as protein denaturation [52]. When most proteins are denatured, their biological function is lost. Rheumatoid arthritis and other rheumatic conditions can produce autoantigens as a result of protein denaturation, a well-established source of inflammation. In vitro research on the processes underlying its anti-inflammatory action has made use of it [53]. Albumin denaturation is one of the preventive effects of polyphenols against protein denaturation. Their capacity to interact with the structure of proteins is the main mechanism behind this activity. By establishing hydrogen bonds with the protein’s amino acid residues through their hydroxyl groups, polyphenols can stabilize the protein’s structure and stop it from losing its secondary and tertiary conformations, which usually happens when it is exposed to heat or other denaturing agents [31,54,55].

#### 3.3.2. Heat-Induced Hemolysis

Figure 3B illustrates a dose-dependent protective effect of SEFE and aspirin against hemolysis. SEFE showed lower efficacy than aspirin, with IC50 values of 87.64 ± 5.35 μg/mL. Phenolic chemicals in the plant extract may have an anti-hemolytic action because they stabilize the membrane of red blood cells, preventing them from lysing. Without changing the fluidity of the hydrophobic portion, they integrate into the erythrocyte membrane’s outer lipid layer, changing the hydrophilic portion’s layout. They shield the cells in this way from harmful chemicals like reactive oxygen. According to studies, adding phenolic compounds, like flavonoids, to erythrocyte membranes increases their resistance to hypotonic lysis. This could be because of the cells’ altered size or shape due to their higher volume/surface ratio. Erythrocytes are more susceptible to hemolysis when exposed to high temperatures because it distorts their membranes. The membrane is shielded from heat by the interaction of polyphenols with membrane proteins, which prevents denaturation [56,57].

#### 3.3.3. Lipoxygenase Inhibition

Figure 3C shows the results of the lipoxygenase inhibition experiment, which assessed anti-inflammatory activity. SEFE significantly inhibited lipoxygenase, with an IC50 value of 146 ± 1.17 μg/mL, but this was still less than the anti-lipoxygenase activity of quercetin with a value of 27.14 ± 0.02 μg/mL. These findings demonstrate a distinct dose-dependent relationship: the percentage of LOX inhibition increases with drug concentration. At every concentration examined, quercetin in particular outperformed SEFE extract in terms of LOX activity inhibition. Quercetin’s chemical structure may be the cause of this, as it may interact with LOX more favorably and inhibit it more successfully. Many illnesses, including cancer, heart disease, strokes, and neurological disorders, can be brought on by the excessive production of inflammatory mediators. Lipoxygenases (LOXs) are monomeric proteins that produce hydroperoxides by oxidizing polyunsaturated fatty acids, particularly arachidonic acid and linoleic acid. LOX products are important in inflammation and can be converted into various compounds. It is consequently possible to control the inflammatory process by regressing LOX activity [14,58].

### 3.4. Analgesic Test

**Figure 4:**
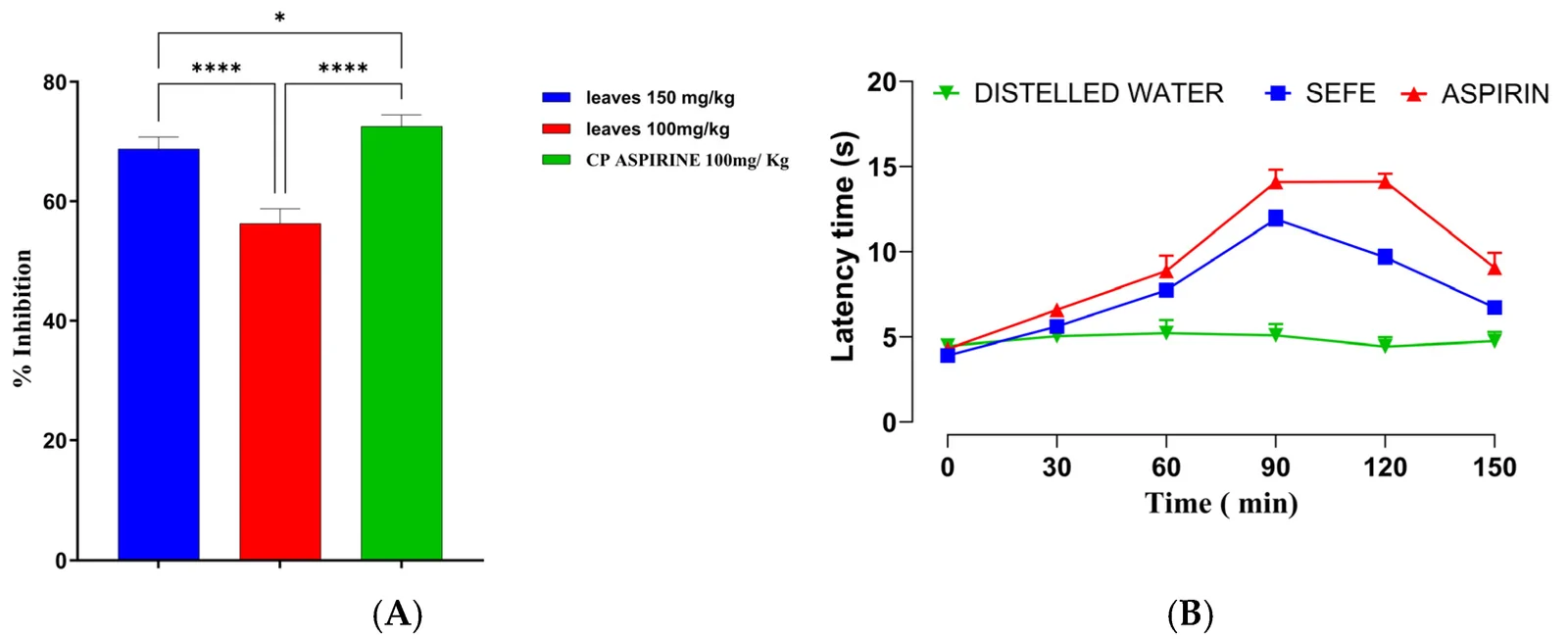
Analgesic effect of SEFE and aspirin, (**A**) Writhing test, (**B**) Tail flick test. Values are expressed as mean ± standard deviation (SD). * *p* < 0.01 compared to the control group; **** *p* < 0.0001 compared to the control group.

Figure 4A,B show the findings of two methods used to investigate the plant extract’s analgesic effect: the withdrawal test and the tail test. When compared to aspirin, a well-known peripheral analgesic, which was utilized as a positive control in this investigation, oral administration of the hydroethanol extract of *S. elaeagnifolium* leaves at a concentration of 150 mg/kg considerably, decreased the nociceptive impact of acetic acid in rats. Moreover, the extract showed a 68.01% torsion inhibition rate compared to 72.16% for aspirin.

Figure 4B displays the tail test findings. This activity is demonstrated by the enhanced response time of rats to radiant heat following 60 min of drug administration: response time for the 150-extract dose was 7.74 after 60 min, whereas aspirin had a little longer time (8.89 s). Even so, the reaction time was still high after 120 min of SEFE administration, with a value of 9.67 for the 150 mg/kg dose, which was marginally less than the aspirin response time of 14.21 s. *S. elaeagnifolium* extract has both a peripheral and central analgesic effect, according to the test results. However, its duration of action is marginally shorter than that of aspirin, especially for the central impact. Whereas the tail test’s increased reaction time suggests an action on central pain pathways, such as receptor modulation or specific neuronal pathways, the withdrawal test’s inhibition of twisting suggests that the extract blocks certain peripheral pain mechanisms, possibly through inhibition of prostaglandins [33,59].

Much research has been done on the analgesic effects of species in the Solanum genus since many of these plants contain bioactive chemicals that may have pain-relieving qualities. Acute, neuropathic, and inflammatory pain are among the many species of the Solanum genus that are used in traditional medicine to alleviate pain [60,61,62]. In one study, *Solanum melongena*’s crude methanolic extract had a potent analgesic effect across a range of tests. It effectively decreased acetic acid-induced nociception at dosages of 10, 20, and 50 mg/kg (*p* < 0.001). In the formalin test, it decreased the mice’s licking response in both phases (*p* < 0.01, *p* < 0.001). Additionally, a dose of 50 mg/kg demonstrated analgesic effects equivalent to those of morphine in the hot plate test (*p* < 0.01, *p* < 0.001) [63]. In two mouse studies, the leaf extract of *Solanum surattense* showed strong analgesic activity it protected mice from thermal pain by 39.34% to 60.47%, reduced acetic acid-induced writhing by 33.98% to 49.59%, and decreased formalin-induced pain by 40.00% to 67.80%. The analgesic effects were considerable at doses of 250 and 500 mg/kg [64].

### 3.5. Molecular Docking

Cyclooxygenase (COX) inhibition involves blocking the activity of COX-1 and/or COX-2, enzymes responsible for converting arachidonic acid to prostaglandins, which regulate inflammation, pain, and fever. COX-2, induced during inflammation, is primarily associated with pain and swelling, and its selective inhibition minimizes inflammation and pain while reducing gastrointestinal side effects.

In our in silico study, all phytocompounds exhibited a remarkable inhibition of cyclooxygenase-2 with a glide gscore merge between −5.698 and −7.469 kcal/mol. Quercetin, catechin, and Syringic Acid were the most active molecules against cyclooxygesae-2 with a glide gscore of −7.469, −7.193, and −6.371 Kcal/mol respectively.

Lipoxygenase (LOX) inhibition involves blocking the activity of LOX enzymes, which catalyze the oxidation of polyunsaturated fatty acids, such as arachidonic acid, to produce leukotrienes and other bioactive lipid mediators. These mediators play a critical role in inflammation, allergy, and oxidative stress-related conditions (Table 4). Regarding our in silico study, Gallic acid, Caffeic Acid, and p-coumaric acid were the most active molecules against lipoxygenase with a glide gscore of −6.784, −6.514, and −6.394 kcal/mol respectively.

Furthermore, the inhibition of glycation plays a significant role in antidiabetic activity by preventing the formation of advanced glycation end products (AGEs), which are formed through the non-enzymatic reaction between reducing sugars and proteins, lipids, or nucleic acids. AGEs accumulate in chronic hyperglycemia and contribute to diabetes-related complications such as neuropathy, nephropathy, retinopathy, and cardiovascular diseases. In our in silico study, Syringic Acid, Gallic acid, and 3-4-dihydroxybenzoic acid showed the highest inhibitory activity against VC1 domain of the receptor for advanced glycation end products with a glide gscore of −5.672, −5.166, and −4.922 kcal/mol.

**Figure 5:**
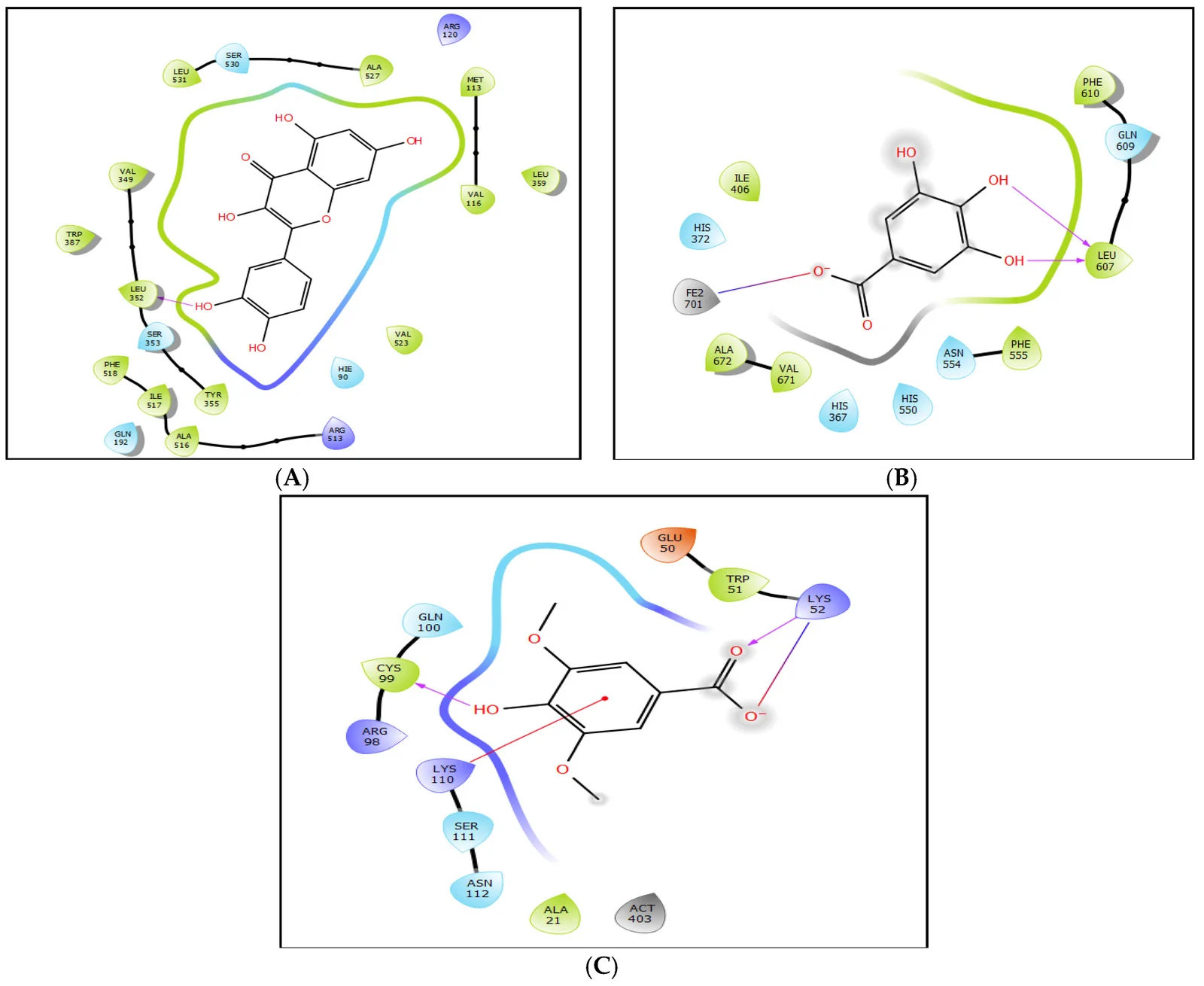
The 2D view of ligand interactions with the active site. (**A**): Quercetin interactions with the active site of cyclooxygenase. (**B**): Gallic acid interactions with the active site of lipoxygenase. (**C**): Syringic acid interactions with the active site of Receptor for Advanced Glycation End Products VC1 domain.

**Figure 6:**
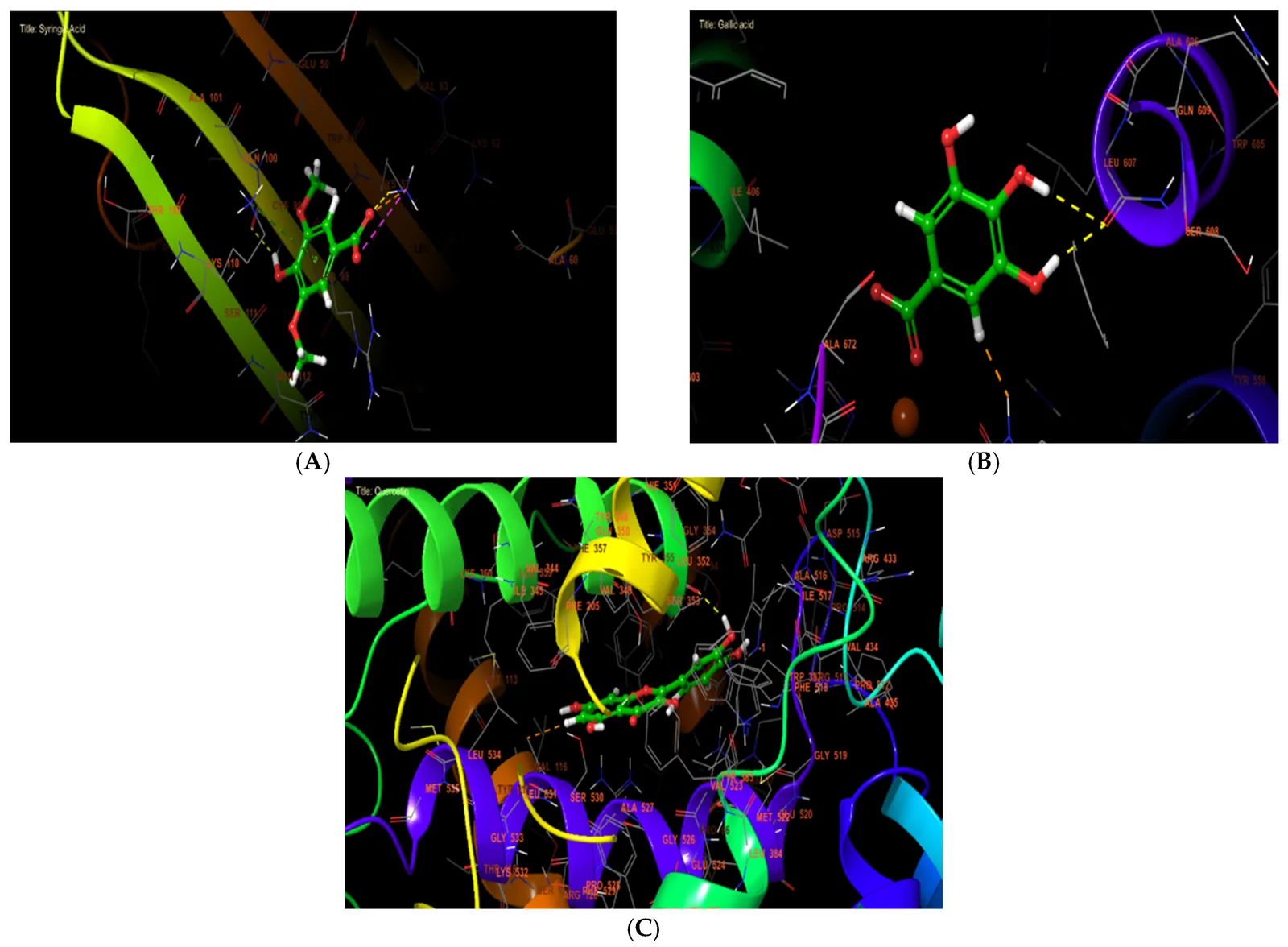
The 3D view of ligand interactions with the active site. (**A**): Quercetin interactions with the active site of cyclooxygenase. (**B**): Gallic acid interactions with the active site of lipoxygenase. (**C**): Syringic acid interactions with the active site of Receptor for Advanced Glycation End Products VC1 domain.

The 2D and 3D interaction analyses between Quercetin and cyclooxygenase active site showed the formation of one hydrogen bond with residue LEU 352 (Figure 5A and Figure 6A). Moreover, gallic acid established two hydrogen bonds with residue LEU 607 and one salt bridge with residue FE2 701 (Figure 5B and Figure 6B).

In the Receptor for Advanced Glycation End Products VC1 domain active site, Syringic acid established two hydrogen bonds with residues LYS 52 and CYS 99, one salt bridge with residue LYS 52, and one Pi cation with residue LYS 110 (Figure 5C and Figure 6C).

## 4. Conclusions

In conclusion, HPLC analysis revealed that the primary phenolic constituents of the extract were 3,4-dihydroxybenzoic acid, caffeic acid, gallic acid, syringic acid, and quercetin. Additionally, ursolic acid was present, a natural triterpenoid renowned for its significant pharmacological properties, including antioxidant, anti-inflammatory, and neuroprotective activities. Furthermore, it exhibited considerable anti-inflammatory activity, as evidenced by its potent inhibition of albumin denaturation, lipoxygenase activity, and hemolysis, with favorable IC_50_ values. Notably, the extract also showed analgesic effects comparable to those of standard reference drugs. These experimental findings were further corroborated by in silico molecular docking studies, which provided mechanistic insights into the observed bioactivities. Collectively, these results suggest that the extract possesses multifaceted therapeutic properties, making it a promising candidate for further development in the management of metabolic and inflammatory disorders.

## Figures and Tables

**Table 1:** Chemical substances present in *S. elaeagnifolium* leaf extracts revealed by HPLC.

Pick Number	Molecules	Retention Time	Area %	Structure
1	Gallic acid	5.45	8.18	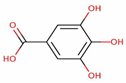
2	3,4-dihydroxybenzoic acid	7.68	4.78	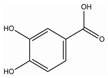
3	Catechin	8.50	6.67	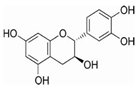
4	Caffeic acid	11.55	1.28	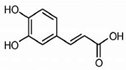
5	Syringic acid	11.98	1.78	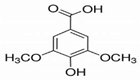
6	*p*-coumaric acid	14.68	20.49	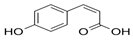
7	Quercetin	30.38	24.09	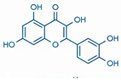
8	Ursolic acid	32.75	2.6	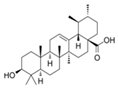

**Table 2:** IC_50_ values of the suppression of albumin glycation ascertained through assessments involving TBA, DNPH and Congo red tests and the formation of AGEs.

	Glycation Inhibition Reaction Assay			
	TBA (mg/mL)	DNPH (mg/mL)	Congo Red (mg/mL)	AGEs (mg/mL)
SEFE	0.51 ± 0.012	0.52 ± 0.005	0.45 ± 0.006	0.49 ± 0.007
AG	0.45 ± 0.006	0.46 ± 0.003	0.46 ± 0.006	0.45 ± 0.005

Values are expressed as mean ± SEM.

**Table 3:** Effects of SEFE membrane stabilization on heat-induced hemolysis test, prevention of albumin denaturation, and inhibition of lipooxygenase.

Inflammatory Inhibition Assay			
	IC_50_ Heat-Induced Haemolysis (μg/mL)	IC_50_ Inhibition of Albumin Denaturation (μg/mL)	IC_50_ Inhibition of Lipoxygenase (μg/mL)
SEFE	87.64 ± 5.35 **	86.94 ± 1.63 **	146 ± 1.17 ****
Diclofenac	-----	76.46 ± 1.94	-----
Aspirin	74.81 ± 3.01	----	-----
Quercétine			27.14 ± 0.02

Values are expressed as mean ± standard deviation (SD). ** *p* < 0.01 compared to the control group; **** *p* < 0.0001 compared to the control group.

**Table 4:** Docking results in ligands in different receptors.

	Glide Gscore		
	6COX—Minimized	3V99—Minimized	7LMW—Minimized
3,4-dihydroxybenzoic acid	−6.071	−6.342	−4.922
Caffeic Acid	−6.096	−6.514	−4.125
catechin	−7.193	−5.681	−4.88
Gallic acid	−5.967	−6.784	−5.166
p-coumaric acid	−5.698	−6.394	−4.107
Quercetin	−7.469	−6.707	−4.895
Syringic Acid	−6.371	−6.333	−5.672

## Data Availability

The data presented in this study are available on request from the corresponding author.
